# Development of a novel ensemble learning model for predicting asphalt volumetric properties using experimental data for pavement performance assessment

**DOI:** 10.1038/s41598-025-26364-8

**Published:** 2025-11-19

**Authors:** Ali Javadzade Khiavi, Babak Naeim, MohammadAmin Soleimanzadeh

**Affiliations:** Department of Civil Engineering, University of Mohaghegh Ardabili, Daneshgah Street, Ardabil, 56199-1136 Iran

**Keywords:** Asphalt volumetric properties, Ensemble learning, Optimum bitumen percentage, Specific gravity of aggregates, Asphalt sample density, Engineering, Materials science, Mathematics and computing

## Abstract

The study is conducted across the board from laboratory experiment to the practical use of a cutting-edge machine learning algorithm, aiming to predict the behavior of asphalt mixtures, the focus being on three aspects: Aggregate Void Percentage (AVP), Percentage of Voids Filled with Bitumen (PVFB), and Percentage of Voids in the Marshall Sample (PVMS). Approximately 200 road surface samples from Ardabil city in Iran were taken and characterized based on 11 influential features, including Optimum Bitumen Percentage (OBP), Specific Gravity of Aggregates (SGA), Asphalt and Ambient Temperatures (Aste, AmTe), Fracture Resistance, Softness, and Density. The work was complemented by employing two regression algorithms, namely XGBoost and LightGBM, on the experimental data to forecast volumetric outputs. To further improve the model’s prediction stability, the ensemble (Voting and Stacking) technique was implemented, while the Artificial Protozoa Optimizer (APO) and Greylag Goose Optimization (GGO) were employed for hyperparameter tuning to achieve better generalization and higher accuracy. To overcome the curse of dimensionality and enable the interpretability of the parameters, the authors first performed feature selection followed by a sensitivity analysis. Results show that XGBoost can offer excellent values for both R^2^ and RMSE for all output variables. The methods of ensemble and optimization are among the factors that further push these values. These results demonstrate the potential of combining traditional pavement experimental data with machine learning algorithms for predicting asphalt mixture performance, hence paving the way for a more cost-effective pavement design and management.

**Supplementary Information:**

The online version contains supplementary material available at 10.1038/s41598-025-26364-8.

## Introduction

### Background and related studies

By definition, accurate, real-time information concerning the current and future physical characteristics of the materials, roadway layers, and infrastructure used is the basic need for best decision-making in road construction works involving asphalts^1^. Most of these characteristics can only be directly measured through highly technical and elaborate processes, which also require very expensive equipment, specialized staff, and a significant amount of working time^2,3^. For instance, the tests on asphalt done in a laboratory or with devices such as a non-nuclear density gauge (NNDG) and a falling weight deflectometer (FWD) are at the core of this category. Even if it is not known exactly how these factors will affect the situation, prediction models trained on potentially relevant input variables may be a step in the right direction^4,5^. This has been strongly supported by recent developments in machine learning (ML), which have been widely applied in the field of asphalt road construction. The field of ML prediction models for physical attributes is rapidly evolving, with a focus on different road layers and construction operations, such as compaction and quality inspection, prior to handover. Prediction models have been introduced to replace the lengthy and tedious experimental activities required for estimating subgrade modulus, California Bearing Ratio (CBR), and asphalt layer density. To facilitate compaction by machine operators, an increasing number of researchers are developing models^6^.

Nevertheless, the plethora of ML methodologies for digitizing past data for model training, preprocessing of input variables, and performance evaluation of predictions makes it extremely challenging to develop efficient prediction models^7,8^. Therefore, the first step should be to recognize and link the information available in research publications to the conceptual framework of ML techniques, thus making the modelling process more convenient. Understanding this mapping is crucial for grasping the current state of the subject and identifying the relevance of ML methods. Moreover, there are very few literature reviews conducted on this topic. These have either concentrated on studies that employ Artificial Neural Networks (ANNs) as the sole technique, disregarding other methods, or studies of road usage focusing on pavement condition, but not on construction. As a result, an additional study is required to assess the accuracy of physical property prediction models^9,10^.

Asphalt performance prediction has been a critical research area, with various studies investigating computational methods for analyzing long-term properties, mechanical strength, and durability. Different research has utilized ML techniques to predict the properties of the asphalt^11–15^. Using Google Street View images, Majidifard et al.^16^ presented a method based on deep learning for automatically identifying distress on pavements. Their work utilized a U-Net model for measuring and segmenting the distress, as well as the YOLO model based on deep learning for classifying the distress. They developed a hybrid system that effectively classified the pavement condition by combining the models, thereby minimizing the need for human intervention and maximizing the accuracy of the surveys. This work demonstrates the increasing applicability of deep learning in pavement appraisal by establishing that image-based analysis can be utilized to create valid pavement condition indices. In another study proposed by Majidifard et al.^17^, ML techniques for the fracture energy estimation of asphalt mixes are provided. Two models were formulated: a Simulated Annealing/Artificial Neural Network hybrid and Gene Expression Programming. An experimental database with disk-shaped compact tension (DC(T)) test results was used to train the models. The models included various predictor factors, which included the test temperature, reclaimed asphalt pavement and shingles (RAP and RAS) content, asphalt content, aggregate size and gradation, asphalt binder performance grading (PG), and crumb rubber content. By predicting the effective asphalt content (Pbe) and the absorbed asphalt content (Pba), Liu et al.^18^ delve into ML to enhance the Superpave mix design and minimize the use of conventional trial-and-error techniques. Five ML models, namely Random Forest, SVR, and Gradient Boosting, were trained on the mix design parameters. The optimum accuracy was obtained with the use of gradient boosting (R^2^ = 0.95). An efficient, automated method for designing the asphalt mixes was demonstrated through a case study that verified that the ML-based prediction closely matched the result obtained using tests.

A systematic analysis presented by Leukel et al.^19^ reviews 30 papers (2011–2023) on ML models for predicting the physical properties in the construction of asphalt roads. The results demonstrate the employment of various input variables and sensors, a low need for feature selection, and the use of ANNs with uncertain advantages in their performance. The work highlights the underutilization of unitless performance metrics and the need for improved reporting on the training/test data. The review gives a complete overview of the application of ML in decision-making in the construction of asphalt roads. Hosseini et al.^20^ developed ML models optimized for viscoelastic property forecasting of modified binders using an experimental dataset acquired by dynamic shear rheometer (DSR) tests at seven temperatures ranging from − 22 to 22 °C. Additives, including crumb rubber, SBS, and PPA, were considered. ML models, including ANN, SVR, Decision Tree, and Ensemble Regression, were trained, and their performances were assessed. The results indicated that Ensemble Regression was the most accurate model to predict the complex shear modulus (G) and phase angle (δ)* in modified binders. Talebi et al.^21^ explore ML models to predict fracture toughness in asphalt mixtures using 675 experimental data points. The Relief technique selects significant input factors, and three ML models, Support Vector Regression (SVR), ETR, and GBR, are optimized using Particle Swarm Optimization (PSO). Ensemble voting and stacking improve predictive accuracy to 91.57%. Individual Conditional Expectation (ICE) plots validate model interpretation, showing the potential of ML to enhance asphalt mixture durability and infrastructure safety.

In addition, recent research continues to demonstrate the growing integration of hybrid and optimization-assisted ML techniques in pavement engineering. For example, Zhang et al.^22^ combined SVR with a Genetic Algorithm to optimize the prediction of asphalt binder viscosity, achieving superior generalization over standalone ML models. Guo et al.^23^ applied ensemble gradient boosting models with Bayesian optimization for predicting rutting and cracking performance indices, emphasizing the need for interpretable and data-driven pavement design. In parallel, several studies have focused on combining ML and metaheuristic algorithms for model optimization and uncertainty reduction. Wang et al.^24^ implemented a particle swarm–optimized extreme gradient boosting model for pavement roughness forecasting and demonstrated a consistent reduction in RMSE compared to the standard XGBoost. Zhang et al.^25^ developed a hybrid prediction model combining Extreme Gradient Boosting (XGBoost) and Whale Optimization Algorithm (WOA) to predict the dynamic modulus of asphalt mixtures. The effects of binder properties, test conditions, mixture volume, and gradation were analyzed. Shapley Additive Explanations (SHAP) quantified variable contributions, and Partial Dependence Plots (PDP) evaluated interactions. Results showed that the binder shear modulus, test temperature, and viscosity were the most influential factors. Optimized binder characteristics, mixture gradation, and test conditions increased dynamic modulus. The study provided a foundation for intelligent asphalt mixture design. These papers collectively illustrate a strong shift in the current literature toward ensemble learning, hyperparameter optimization, and explainable AI, confirming the importance and timeliness of the present study’s integrated approach.

### Contributions and objectives

Despite these advancements, current literature still lacks models developed from comprehensive experimental datasets that simultaneously incorporate ensemble learning, optimization, and feature interpretability. In response to this gap, the present research proposes a novel hybrid framework that integrates advanced boosting algorithms with metaheuristic optimization and feature selection to improve the predictive accuracy of asphalt volumetric properties. Existing literature on the prediction of asphalt performance has focused predominantly on the application of ML-based techniques for the detection of distress, fracture energy, and mixture design optimization based on images. The majority have employed ML models, but few have incorporated ensemble learning, advanced hyperparameter optimization, or rigorous feature selection. Few publications have also designed their own experimental datasets to develop interpretable and generalizable ML models for the prediction of asphalt performance. New contributions of this research:

- *Complete Experimental Dataset*: Unlike previous studies that relied on limited or publicly available datasets, this study collected approximately 200 asphalt samples from Ardabil roads over an 18-month period, creating a diverse and representative dataset with 14 critical features that affect asphalt performance.
- *Integration with the latest ML models*: Whereas earlier work had employed individual ML models, the current paper utilizes XGBoost and LightGBM, two of the most powerful gradient boosting algorithms recognized for their high performance in structured data problems.
- *Advanced Hyperparameter Optimization*: Utilize APO and GGO to optimize model hyperparameters, thereby increasing efficiency and generality —a step that has traditionally been omitted in previous work.
- *Ensemble Learning for Better Performance*: In contrast to previous work that relied mostly on individual ML models, this study employs voting and Stacking ensemble techniques to enhance predictive accuracy, demonstrating their utility in forecasting asphalt performance.
- *Feature selection and interpretability*: Employing feature selection techniques to identify the most significant variables to make the model transparent, reduce computational costs, and enhance interpretability. Sensitivity analysis is conducted to ascertain the significance of the attributes and their contribution towards model prediction.
- *Benchmarking Against Traditional Approaches*: Compared with other research on the application of ML, the outputs are cross-validated against conventional techniques for measuring the performance of asphalt, demonstrating the superiority of the ML models in terms of accuracy, efficiency, and stability.
- *Practical Implications for Infrastructure Durability*: The results show that predictive models based on ML can enhance decision-making in road building and upkeep, providing cost-effective and durable infrastructure solutions.

**Fig. 1:**
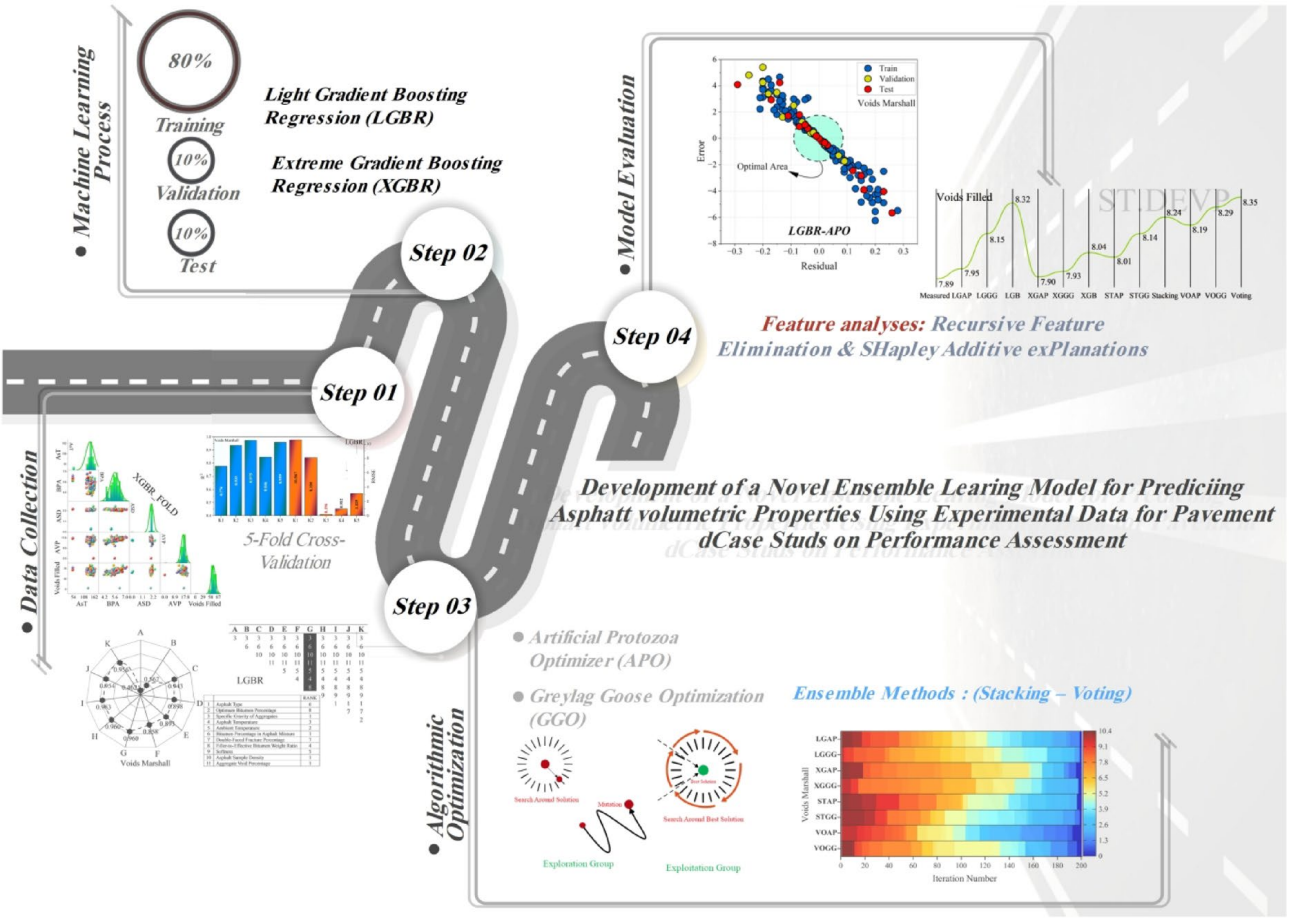
Process of the present study.

This research fills the gap that exists between experimental asphalt performance analysis and current ML methods. This research surpasses the limitations found in current research by proposing a new dataset, utilizing optimization-based learning, and enhancing interpretability through ensemble methods and feature selection. This research proves to be more accurate and robust, making ML-based models suitable alternatives to conventional methods for testing asphalt, leading to more intelligent, data-driven infrastructure planning in the long run. Figure 1 shows the process of the present study.

## Materials and methods

### Overview of the study process

**Fig. 2:**
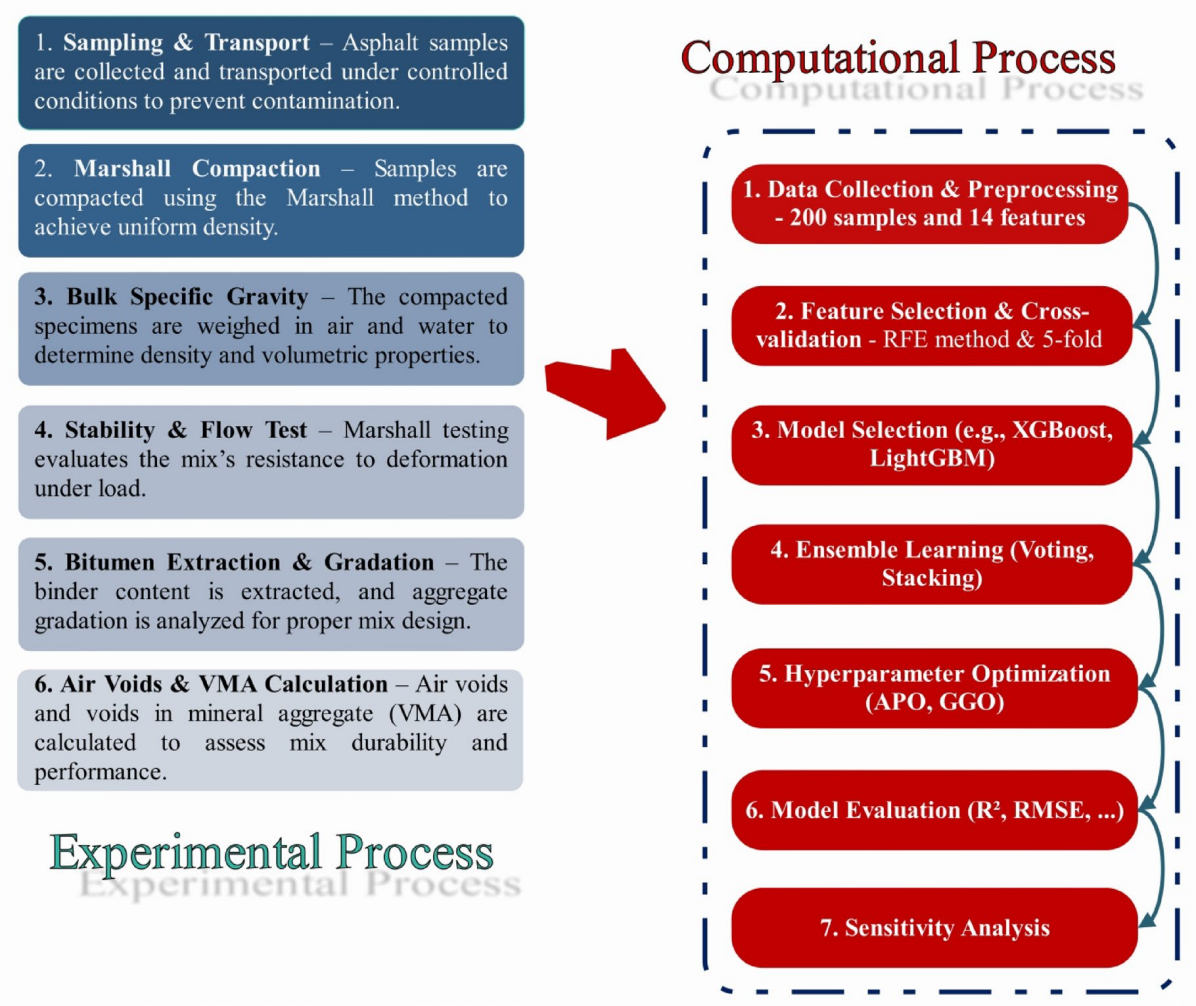
An overview of the process was conducted in this research.

The research was conducted using approximately 200 samples of asphalt, collected from the streets of Ardabil City, Iran, between June 2023 and November 2024. Data preprocessing was performed before implementing ML models to fill in missing values, normalize numerical variables, and encode categorical variables, ensuring the data was more reliable and consistent. ML algorithms, specifically LGBR and XGBR, were effectively utilized to develop a robust prediction model. These models were identified as most suitable in this case due to their ability to handle complex feature interactions and their superior predictive accuracy. In addition to the individual models, ensemble learning techniques were also employed to enhance prediction accuracy. The Voting method merged the outputs of different models to facilitate generalization, while the Stacking method allowed a meta-model to gain maximum predictive power by combining the outputs of base learners. RFE feature selection was employed to positively impact the models, increase their interpretability, and identify the most prominent variables while reducing computational complexity. Moreover, hyperparameters were also tuned to achieve several purposes, such as improved efficiency and better generalizability of the models. Additionally, the APO and GGO methods were implemented to aid in model refinement, thereby facilitating the best possible outcomes through accelerated convergence rates and minimized error. Model validation procedures, such as k-fold cross-validation, were employed to ensure the model’s robustness and prevent overfitting. The accuracy and stability of the model were measured using the coefficient of determination (R^2^) and the Root Mean Square Error (RMSE). A sensitivity analysis was also conducted to assess the relative importance of input variables in model prediction, identifying the most influential sources of asphalt performance. Figure 2 illustrates the flowchart outlining the study process, including the sequential steps taken in the present work.

### Data collection and experimental process

**Fig. 3:**
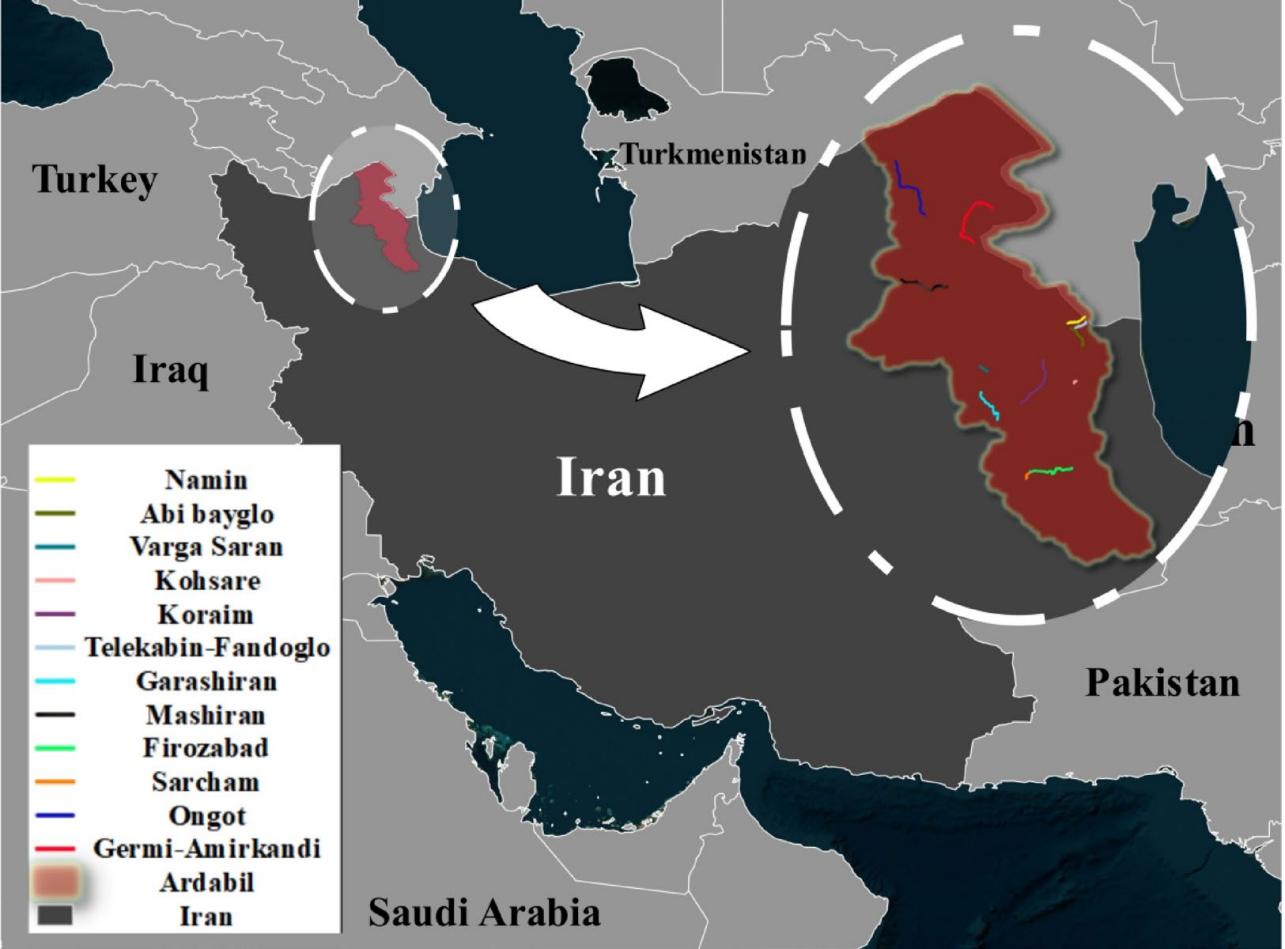
Locations of Collected Samples in Ardabil, Iran.

To develop a robust ML model for the prediction of the properties of asphalt, a large dataset was collected in the area of Ardabil, Iran, from June 2023 to November 2024. The dataset comprised approximately 200 samples of asphalt, representing a vast range of asphalt conditions, composition, and environmental conditions. The dataset comprised various roads, including Dabanlou, Sarcham, Vargeh Saran, Namin, Abibiglou, Alani Streets, Fakhrabad Streets, Ardabil-Sarcham, Ghara Shirian, Viretsk, Dabanlou, Dash Balagh, Jagh Jagh, Shahrak Sanati Streets, Gali Balagh, Meshiran-Meshiran, Shablu-Kohsara, Cable Car to Fandaghloo, and Vegetable Oil Factory Road. Figure 3 shows the locations of collected samples.

#### Experimental process

##### Sampling and transportation of hot asphalt to the laboratory

For the validity and accuracy of the samples collected, the sampling process adhered to the AASHTO T168-03 standard. The process involved field inspections of active asphalt areas and sampling of representative asphalt. The samples were split into three equal test specimens, weighing a total of at least 1200 g per sample. The specimens were stored in metallic containers to facilitate transportation. In the laboratory, the samples underwent quality control and splitting in accordance with the AASHTO T248. They were then loaded into an oven set to DIN 12,880 standards and heated to the desired mixing temperature (135–140 °C). The specimens were then loaded directly into cylindrical Marshall molds with a fixed base plate to prevent material loss.

##### Marshall compaction process

The asphalt samples were compacted according to the traffic load specification and asphalt type using a calibrated Marshall hammer. The hammer was dropped 18 inches to provide a specified number of strikes on both sides of the sample, per standard compacting procedures outlined in AASHTO T245-97 (2004). The molds were stored at room temperature for a period of at least four hours, until they reached a temperature of 25 ± 5 °C. A SPECIMEN EXTRACTOR extracted the compacted specimens**.**

##### Determination of bulk specific gravity

For the bulk-specific gravity determination of the asphalt samples, the procedure followed AASHTO T166-07 and ASTM D2726. The test involved, first, weighing the dry sample at 25 ± 5 °C (Weight A). Secondly, the specimen was immersed in water for 3–5 min, and its weight was recorded when immersed (Weight C). Third, remove the specimen from the water, dry the surface within 5 s, and weigh the surface-dry weight (Weight B). Finally, calculating the bulk specific gravity with the equation:

1 $$G_{mb} = \frac{A}{B - C}$$

where, A is the mass of the dry specimen in the air (grams), B is the mass of the saturated surface-dry specimen in the air (grams), and C is the mass of the specimen in water (grams). Using this bulk specific gravity, the Marshall sample density was calculated based on water density at 25 °C (0.997 g/cm^3^ or 62.24 lb/ft^3^).

##### Marshall stability and flow test

The Marshall samples were tested for deformation resistance and stability according to AASHTO T245-97 and ASTM 1559. The test entailed placing the specimens in a 60 ± 1 °C temperature water bath for 30–40 min, then washing the test apparatus and the guide rods. The specimens were then placed in the Marshall test machine, where a deformation rate of 2 inches per minute was applied uniformly. The maximum load applied at failure was recorded in the test, and the flow value, i.e., deformation at maximum load, was recorded using a dial gauge. The final values of stability were then determined using correlation coefficients of standard tables.

##### Bitumen extraction and gradation analysis

Analysis of the bitumen in the samples was conducted using the AASHTO T164-05 and ASTM D2172 methods. The procedure involved dissolving the sample in an appropriate solvent, followed by centrifugation to separate the bitumen from the aggregates. The aggregates were dried, and the bitumen was determined by determining the weight difference. Finally, a sieve analysis of the extracted aggregates was conducted to evaluate their gradation.

##### Determination of air voids and voids in mineral aggregates (VMA)

The air void content and VMA were calculated based on bulk and theoretical maximum specific gravity values (AASHTO T209). The formulas used were:

2 $$Air void = \frac{{G_{mm} - G_{mb} }}{{G_{mm} }} \times 100$$

3 $$VMA = \frac{{V_{aggregate} - V_{bitumen} }}{{V_{aggregate} }} \times 100$$

where, $G_{mm}$ is the theoretical maximum specific gravity, $G_{mb}$ is bulk specific gravity, $V_{aggregate}$ showcasing the volume of agegates, and $V_{bitumen}$ is the volume of bitumen.

#### Data description

The dataset comprises 14 significant factors that have a substantial impact on asphalt performance. They comprise several physical and chemical properties of the asphalt mixture, including material, environmental conditions, and structure. The data set contains asphalt type, optimum bitumen percentage (OBP), specific gravity of aggregates (SGA), asphalt temperature (Aste), and ambient temperature (AmTe), which influence the stability and durability of the mix. Additionally, the bitumen percentage in the asphalt mixture (BPSM), double-faced fracture percentage (DFFP), and filler-to-effective bitumen weight ratio (FEBWR) influence the binding nature and structure of the asphalt. Physical and volumetric properties such as asphalt sample softness (Sof), asphalt sample density (AsD), voids in aggregate (AVP), voids filled with bitumen (PVFB), and voids in the Marshall sample (PVMS) indicate the hardness and compactness of the asphalt samples. Table 1 presents a statistical summary of the variables, i.e., mean, median, standard deviation, maximum and minimum values, skewness, and kurtosis. It is evident from the data that high variability is present in certain properties, specifically the specific gravity of aggregates and asphalt temperature, which exhibit large values of skewness and kurtosis.

**Table 1:** Asphalt properties were collected from samples in Ardabil, Iran, along with their statistical overview.

*Statistical Metrics*	Input variables										Output variables	
	OBP	SGA	Aste	AmTe	BPSM	DFFP	FEBWR	Sof	AsD	AVP	PVFB	PVMS
*Median*	5.6	2.639	144	23	5.245	98	0.99	3.4	2.323	16.2	68.2	5
*Max*	6.1	2.702	168	36	6.84	100	1.63	4.5	2.641	19.9	89.7	9.4
*Min*	0.6	2.472	120	9	3.78	85	0.62	2.3	0.289	0.2	4.9	1.3
*St.D*	0.634	0.055	10.652	5.841	0.608	2.417	0.202	0.390	0.209	2.045	9.280	1.321
*Kurtosis*	22.116	1.595	− 0.225	− 0.759	− 0.314	3.766	− 0.458	0.535	80.115	22.348	15.227	1.290
*Mean*	5.373	2.619	143.669	23.768	5.239	97.381	1.018	3.418	2.295	15.985	68.237	5.051
*Skeweness*	− 3.512	− 1.568	− 0.018	− 0.049	− 0.228	− 1.694	0.331	0.147	− 8.634	− 3.441	− 2.234	− 0.018

*Asphalt properties and their corresponding abbreviations: OBP, Optimum Bitumen Percentage; SGA, Specific Gravity of Aggregates; Aste, Asphalt Temperature; AmTe, Ambient Temperature; BPSM, Bitumen Percentage in Asphalt Mixture; DFFP, Double-Faced Fracture Percentage; FEBWR, Filler-to-Effective Bitumen Weight Ratio; Sof, Softness; AsD, Asphalt Sample Density; AVP, Aggregate Void Percentage; PVFB, Percentage of Voids Filled with Bitumen; PVMS, Percentage of Voids in the Marshall Sample.

The raw dataset (in the supplementary document) included some samples with missing or non-standard values, such as Marshall stability tests conducted at non-standard ‘stiffened’ temperatures. To maintain a consistent and representative dataset for modeling, missing or anomalous values were replaced using the mean of the corresponding feature. This approach mitigates potential biases while preserving the overall distribution of the data.

### Machine learning models

#### Light gradient boosting regression (LGBR)

LightGBM is an advanced gradient boosting algorithm that enhances the efficiency and accuracy of decision tree learning. It is efficient in handling large datasets with minimal computational cost by optimizing the allocation of memory and training time, resulting in high predictive accuracy. LightGBM builds models by ensembling regression trees. Unlike other boosting methods that perform level-wise tree growth (such as XGBoost), LightGBM employs a leaf-wise tree growth method with depth restrictions. That is, instead of growing all branches at the current level simultaneously, LightGBM selects the leaf with the largest potential to minimize loss and grows the tree from it. This approach yields faster convergence and improved accuracy compared to level-wise methods. It also increases the risk of overfitting, which LightGBM mitigates by imposing tree depth constraints^26–28^.

##### Histogram-based learning for memory efficiency

One of the strongest aspects of LightGBM is its feature binning, which is based on a histogram. Instead of dealing with unprocessed continuous values, the model bins numerical features into a fixed number of bins. This keeps the memory requirements low, as it allows LightGBM to store data in 8-bit integers rather than full floating-point values. The approach also enhances training speed by eliminating the need for pre-sorted data and reducing the number of potential split points in decision trees. While it simplifies the process, histogram binning does not sacrifice the model’s predictive ability, but rather enhances computational efficiency.

##### Loss function optimization and tree splitting

LightGBM minimizes a defined loss function by adding trees incrementally and optimizing the split. During each iteration, it adds a new tree to the model, updating the forecasts by learning from past mistakes. It does so using the assistance of gradient-based optimization techniques, where LightGBM initially calculates first- and second-order gradients (i.e., the derivatives of the loss function) to arrive at the optimal possible split points. The split selection rule is guided by a trade-off between minimizing the error and preventing overfitting. LightGBM determines an ideal weight for each tree leaf based on the gradient information, allowing the model to learn patterns in the data while maintaining generalizability.

##### Overfitting prevention and regularization

Although the leaf-wise tree growth approach improves accuracy, it forms trees that overfit the training set. LightGBM addresses this problem by incorporating regularization techniques, which include: 1. Depth maximum constraints to prevent trees from becoming excessively large. 2. L1/L2 regularization to prevent over-complex models. 3. Gradient-based early stopping, where training is halted when improvement on validation data stops.

### eXtreme gradient boosting regression (XGBR)

Extreme Gradient Boosting (XGBoost) is a complex gradient-boosting-based ensemble learning algorithm. It is specifically designed to improve classification and regression on structured and tabular data. Deep learning models excel on unstructured data, such as text and images, whereas decision tree-based methods like XGBoost perform well on structured data. XGBoost creates decision trees using a gradient descent algorithm, optimizing predictions by minimizing residual errors at each step. Residuals, the difference between actual and predicted values, play a central role in tree threshold adjustment. The first tree begins with a root node containing all residuals, and further branches develop through the optimization of split points. Similarities are allocated to every decision tree in XGBoost using residual values and a cover term, which is the probability of a leaf. Finding the gain is a key area of optimization, as it measures the improvement when a node is split. It is computed by comparing the similarity of the root, left, and right nodes so that the most informative split is obtained^29–31^.

#### Improvements and optimizations in XGBoost

XGBoost employs several optimizations to enhance computational efficiency and predictive power, distinguishing it from other standard gradient boosting practices. They include:

- *Parallelization*: XGBoost achieves accelerated model training by parallelizing tree construction, allowing multiple computations to occur simultaneously.
- *Depth-First Tree Pruning*: Unlike other methods that build trees level by level, XGBoost employs a depth-first approach, which is less memory-intensive and more efficient.
- *Hardware Optimization*: Internal buffers in each processing thread store the statistics of gradients to minimize computation time.
- *Regularization*: XGBoost incorporates both L1 (LASSO) and L2 (Ridge) regularization to prevent overfitting and improve sparsity.
- *Data Management*: The algorithm effectively handles sparse data by accounting for missing values and utilizing efficient split-finding techniques.
- *Weighted Quantile Sketch*: XGBoost employs this method to identify the optimal split points on weighted data, thereby enhancing feature selection.
- *Automatic Iterative Boosting*: The model incorporates built-in cross-validation, eliminating the need to define the iterations of boosting in advance, and thus offering optimal performance.

#### Artificial protozoa optimizer (APO)

Binary fission is how Euglena reproduces, a simple asexual process where one organism is split into two identical offspring. It is performed optimally at temperatures between 20 and 35 °C. It is initiated when the replication of essential organelles, such as the flagellum, esophagus, and stigma, follows the mitosis of the nucleus. The cell then divides along its axis, starting with a division at the head that progresses backward until the two new organisms are completely separate. The APO algorithm is designed to locate minimization solutions by encoding possible solutions as protozoa. Each protozoan is defined by several variables corresponding to where it is within a search space^32–34^.

- *Foraging Behavior:* Foraging is influenced by both internal and external factors. Internally, protozoa determine their direction based on their energy needs. Externally, environmental conditions of competition and encounters with other organisms influence their foraging.
- *Autotrophic Mode:* Protozoa with chloroplasts can perform photosynthesis to create energy. They will move towards areas of optimal light intensity, avoiding areas that are too intense or too dark. In the simulation, this is represented by moving each protozoan based on environmental inputs and the presence of surrounding protozoa.
- *Heterotrophic Mode:* Protozoa consume organic matter in the environment when it is dark or when it is not possible to carry out photosynthesis. The algorithm mimics this by moving protozoa to regions where food is expected. It is driven by environmental solutions that enable efficient searching and adjustment.
- *Dormancy:* In unfavorable conditions, some protozoa fall into a dormant state to conserve energy. In APO, when a protozoan is inactive, it is replaced by a new one to preserve population balance. It prevents the stagnation of the optimization process and maintains diverse solutions.
- *Reproduction:* Protozoa reproduce through binary fission when they grow to a suitable size. The algorithm imitates this by duplicating a protozoan and adding slight perturbations to create variants. The mechanism enables exploration of the search space without losing good solutions.
- *Algorithm Integration*: The APO accomplishes this by assigning probabilities to autotrophic and heterotrophic behavior, dormancy, and reproduction. The relative proportion of these processes is an effective optimization method. The only specific parameters that require input are the maximum dormancy and reproduction fractions, as well as the number of neighboring pairs when interacting.

#### Greylag goose optimization (GGO)

**Fig. 4:**
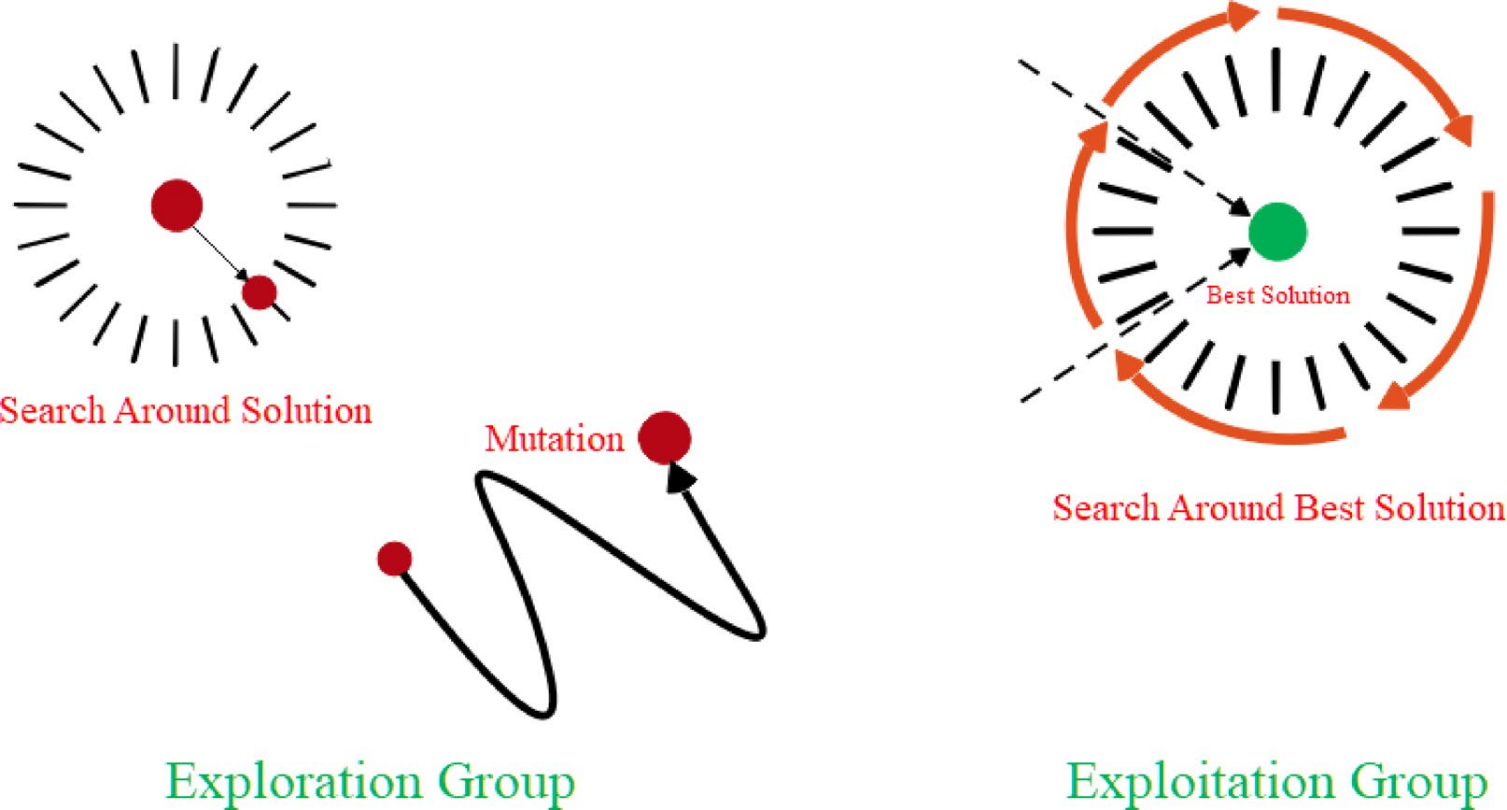
The GGO exploration and exploitation groups.

GGO is a population optimization algorithm inspired by the behavior of the greylag geese. The algorithm begins by generating individuals that represent potential solutions to a problem. Each individual is a potential solution, and the algorithm continually refines the solutions by exploring and exploiting them. Figure 4 illustrates the dynamics of the GGO algorithm, which is both capable of finding new solutions and optimizing current ones. The balance between exploration (discovering new solutions) and exploitation (optimizing current solutions) allows the algorithm to explore the solution space efficiently^35^.

*Exploration Operation*: The search phase of GGO plays a critical role in obtaining the optimal solutions without being trapped in local optima. The algorithm directs individuals to explore promising areas in the search space, thereby avoiding stagnation. For better exploration, the algorithm incorporates a mechanism whereby the geese serve as search agents and search for better positions than their current ones. Additionally, a set of randomly selected agents (paddles) further refines the search by exploring other positions in the search space. The movements and interactions of agents facilitate a diversified approach to exploration. One of the components of the exploratory phase is dynamic parameter tuning that controls motion. Such tuning achieves a balance between extensive explorations at the beginning of the process and subsequent fine-tuning, thereby maximizing the algorithm’s efficiency in converging to optimal solutions.

*Exploitation Operation*: The exploitation phase is devoted to improving existing solutions by evaluating and optimizing them based on their performance. The better-performing solutions have a higher priority for implementation by GGO. This phase relies on directing less competent individuals towards more competent ones by employing a set of better solutions, known as sentries. Sentries act as markers, directing other individuals to better positions. The population updates positions by averaging the improvements made through movements, resulting in gradual convergence to an optimal solution.

*Venturing into the Neighborhood of the Optimal Solution*: For further fine-tuning of the solutions, GGO encourages individuals to explore the neighborhoods of the then-best-known solution. This enables fine-tuning, increasing the final accuracy of the solution. The size of this local search is dynamically managed to prevent it from concentrating too heavily on a single area without compromising overall diversity.

*Choice of the Optimal Solution*: GGO employs a mutation mechanism and systematic evaluation of search agents to maintain a high explorative capability. The algorithm periodically rearranges agents within the search space in a way that dynamically redistributes the exploration and exploitation roles. In the final phase, GGO selects the optimal solution that is found over its iterations. It prevents premature convergence and makes the algorithm converge to an optimal solution efficiently.

#### Ensemble methods (stacking – voting)

*Stacking Ensemble Method*: Stacking is a complex ensembling approach where multiple base models are stacked to develop a stronger and more accurate predictive model. Stacking differs from averaging or voting, as it employs a meta-model that learns to blend the predictions of the base models in the most effective manner. It begins by training several distinct ML models (such as decision trees, support vector machines, or neural networks) on the same dataset. The models predict, and then those predictions get input into the meta-model. The meta-model, often a higher-level model like logistic regression or another ML algorithm, learns to combine the output of the base models to predict the outcome. Stacking enhances predictive accuracy by aggregating the strengths of all the component models. It reduces the errors and biases of individual models by allowing the meta-model to weigh their contributions appropriately. Stacking is especially useful when multiple models identify distinct patterns in the data, resulting in a more generalized and improved prediction^36^.

*Ensemble Method Voting*: Voting is a simpler aggregation method that employs a straightforward decision rule to combine the forecasts of multiple models. It is of two major forms: 1. Hard Voting: All models make a prediction, and the final output is a decision based on a majority vote (in classification tasks). The final prediction is the class voted for by the majority of models. 2. Soft Voting: Soft voting employs not just the last class label, but instead utilizes the class’s predicted probabilities. The model decides with the highest average probability per class. Ensemble voting is particularly effective when the underlying models perform similarly but make different errors. Their voting averages out the inconsistency, yielding higher accuracy and reliability. It is widely used in classification problems, offering a well-balanced decision-making process by aggregating the knowledge of several models. Both stacking and voting help improve ML performance by aggregating multiple models; however, stacking is more complex, as it requires training a meta-model to make the final predictions. Voting is a less complex yet effective approach that relies on either majority voting or averaging probabilities^37^.

## Results and discussion

### Feature selection analysis

**Fig. 5:**
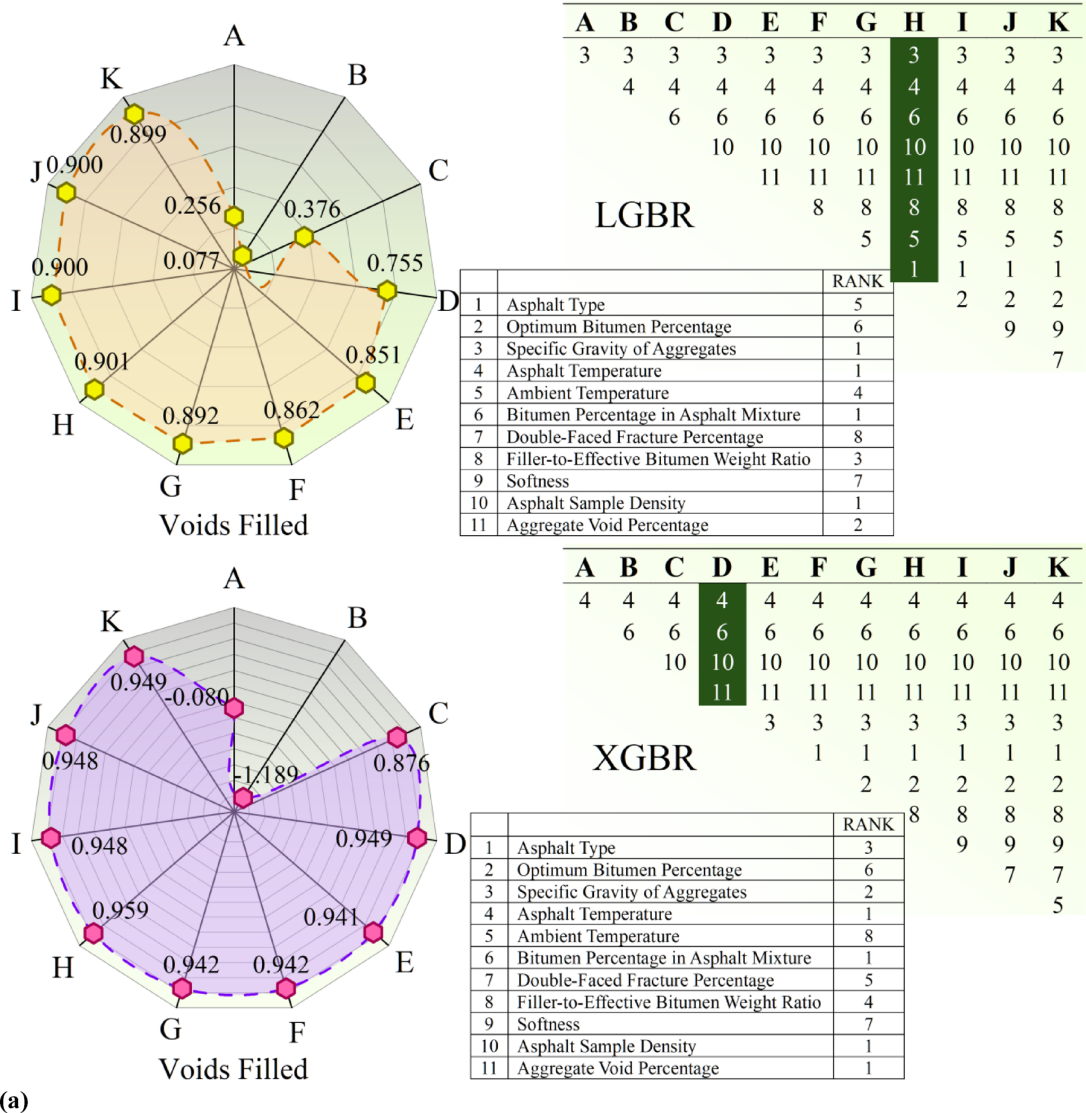

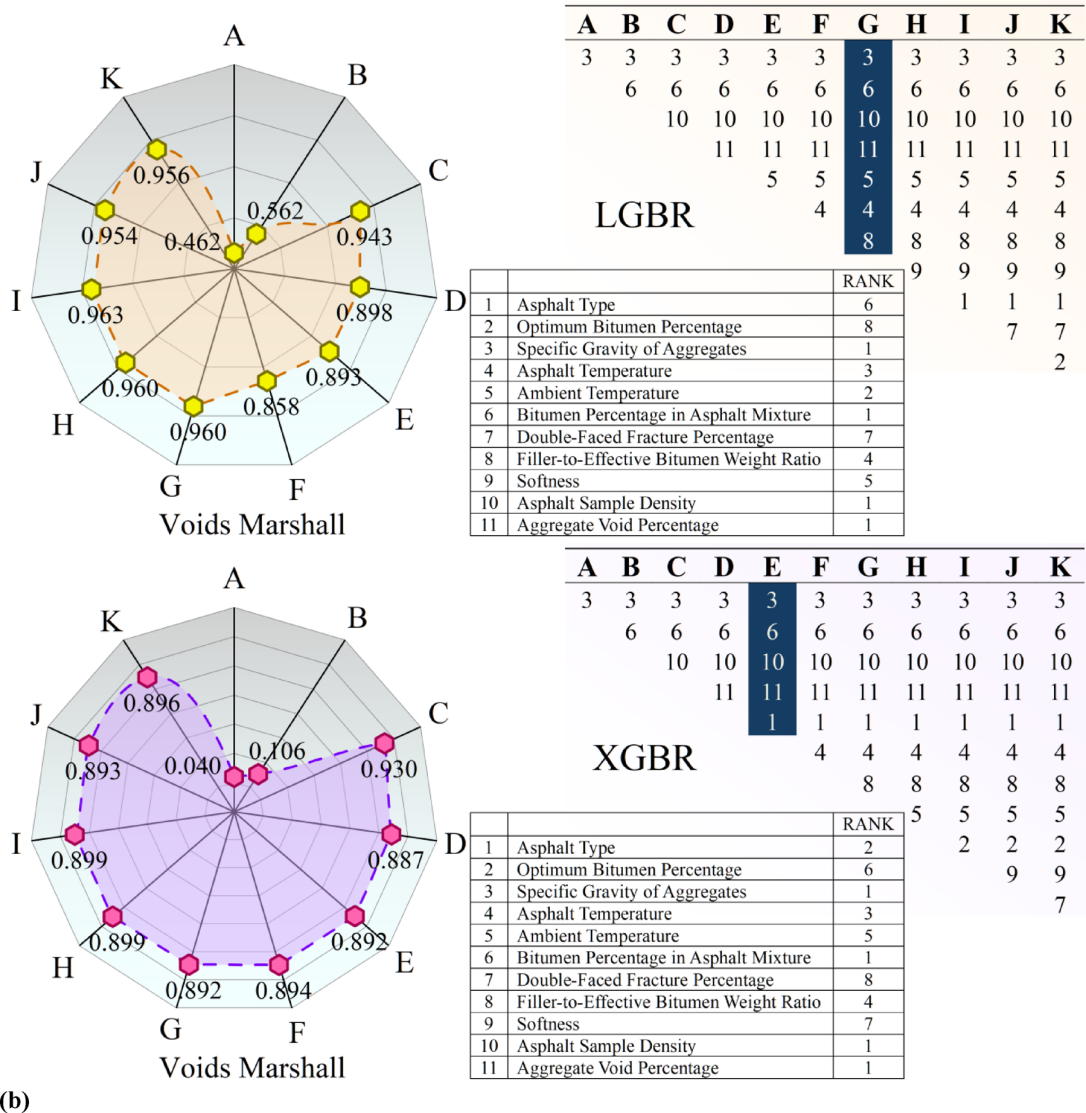
RFE-based feature selection applied to the experimental asphalt dataset, considering developed models in both (**a**) void-filled and (**b**) void-marshall targets.

Figure 5 graphically illustrates the result of Recursive Feature Elimination (RFE) on the asphalt dataset, focusing on two variables: Voids Filled, the ratio of voids in the aggregate structure filled with bitumen, and Voids Marshall, the ratio of air voids in the compacted asphalt sample, an important factor in durability and performance. The ranking of feature importance is illustrated in tables per subfigure, demonstrating how features (A-K) rank in order of importance as defined by RFE. The rankings differ between the Voids Filled and Voids Marshall targets, indicating that features impact these properties to varying degrees. The rankings differ between the XGBR and LGBR models, as these models assign different weights to features. Radial plots graphically represent feature importance, with the relative importance of each feature indicated by the distance from the center. The plots allow easy comparison of feature importance per model and target variable, with values represented as the model’s predictions. The figure also shows a comparison of XGBoost (XGBR) and LightGBM (LGBR) performance, demonstrating differences in feature rankings and patterns in the radial plot, indicating that the models perceive feature importance differently. The tables present a point of comparison that illustrates how each model ranks features, highlighting differences in feature selection. The RFE process is meant to identify the most important features in predicting Voids Filled and Voids Marshall. From the rankings and the radial plots, researchers can observe which variables have the strongest influence.

### Cross-validation results

**Fig. 6:**
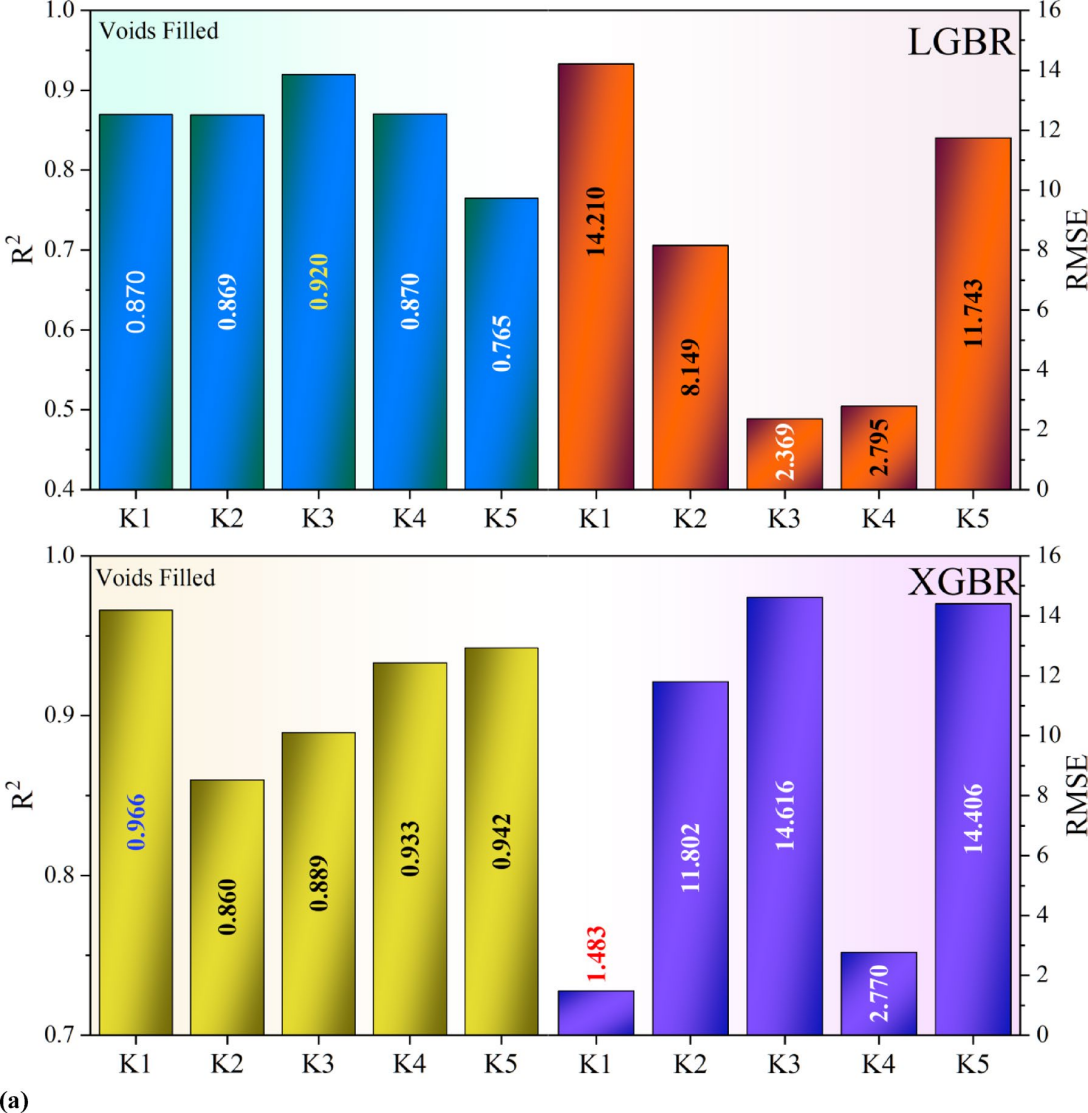

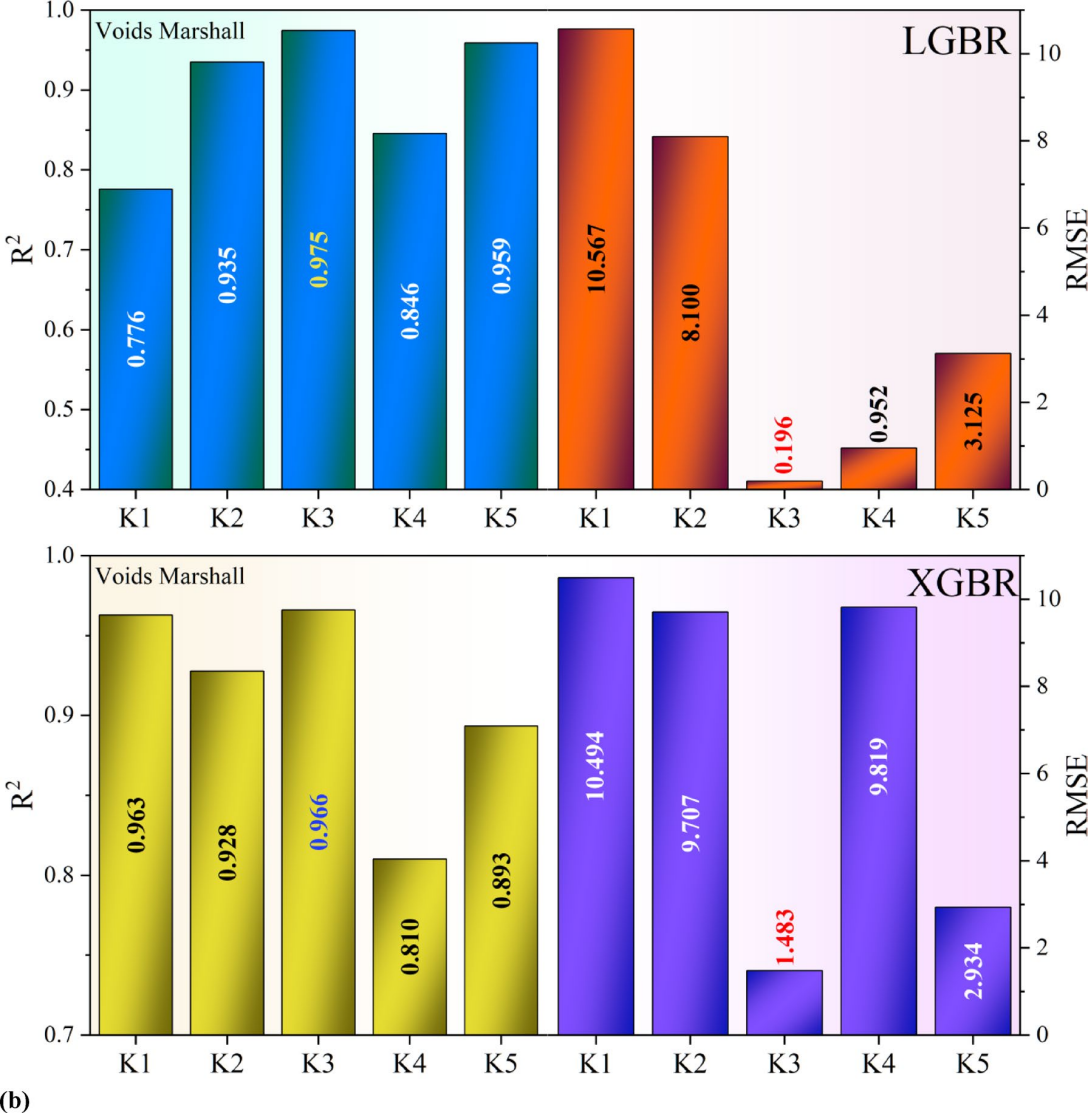
Performance of XGBR and LGBR Models in Predicting (**a**) void-filled and (**b**) void-marshall targets, using 5-Fold Cross-Validation and demonstrating model consistency and performance across different data partitions.

Figure 6 presents the result of the cross-validation conducted on the models. LGBR and XGBR k-fold cross-validation results provide the performance of both models based on R^2^ and RMSE values across five folds. The R^2^ values of LGBR range from 0.7647 to 0.9196, exhibiting some deviation but overall demonstrating good predictive power. XGBR, on the other hand, has a better average R^2^, ranging from 0.8598 to 0.9660, indicating better generalization across the folds. Generally, XGBR outperforms LGBR in terms of R^2^, particularly in Fold 1 (0.9660 vs. 0.8696) and Fold 4 (0.9329 vs. 0.8704), indicating higher predictive power. However, in Fold 3, LGBR has a better R^2^ (0.9196 vs. 0.8893), showing it is better in certain scenarios. The RMSE values of LGBR vary widely, ranging between 2.36 and 14.21, indicating inconsistent predictive errors across the various folds. The XGBR model, however, exhibits a stable trend in RMSE values, with most values being less than 15, except for Fold 3 and Fold 5, where the RMSE is significantly higher. The best performance of XGBR is observed in Fold 1, where it achieves the highest R^2^ (0.9660) and the lowest RMSE (1.48), indicating the model’s optimal performance. The worst performance of LGBR is observed in Fold 5, where it achieves the lowest R^2^ (0.7647) and the highest RMSE (11.74), indicating poor predictive power in that partition. In short, XGBR is better than LGBR in terms of consistency of R^2^ and RMSE and is, therefore, the preferred model choice for this dataset. However, LGBR remains competitive in some folds and could be applied when computational efficiency is a concern.

**Fig. 7:**
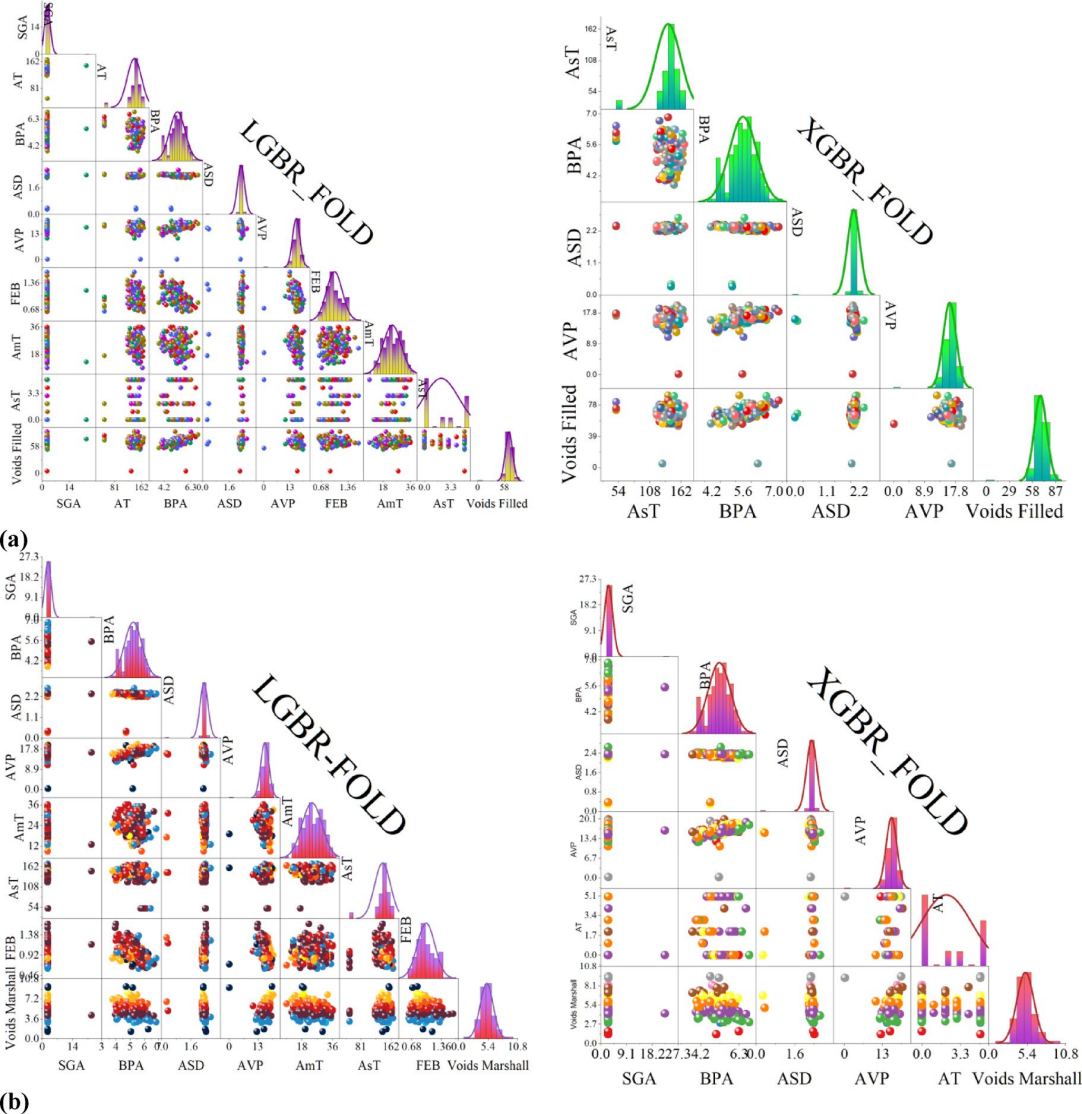
Correlation between the input variables and (**a**) Voids Filled and (**b**) Voids Marshall after feature selection and k-fold cross-validation, representing the final dataset used for model development.

Figure 7 presents correlation matrices and scatter plots illustrating the correlation between input and target variables, Voids Filled and Voids Marshall, after feature selection and k-fold cross-validation. The correlation matrices, illustrated in the upper triangles of each subfigure, reflect the correlation between pairs of input variables, where the color intensity represents the direction and strength of the correlation. The scatter plots in the lower triangles represent the correlation, allowing patterns and trends in the data to be detected. The diagonals of the matrices contain histograms or density plots that represent the distribution of individual variables, providing information on their spread and shape. The figure is split into two columns, one for LGBR-FOLD and the other for XGBR-FOLD, to enable a direct comparison of how each model represents the relations between variables. Subfigure (a) is devoted to Voids Filled, and subfigure (b) is devoted to Voids Marshall, with the latter indicating the various factors influencing the two asphalt properties. The patterns of correlation differ slightly between LGBR and XGBR, indicating the unique way in which the models represent the relationships between variables based on the differences in their learning mechanisms. This is expected as different ML methods prioritize features differently based on their intrinsic structures. The input-target relations also differ, suggesting that the variables influencing Voids Marshall and Voids Filled may not be the same. One input variable could be strongly correlated with one target variable and less so with the other. Since the results follow feature selection, the resulting correlations reflect the relations between the selected variables.

### Convergence analysis

**Fig. 8:**
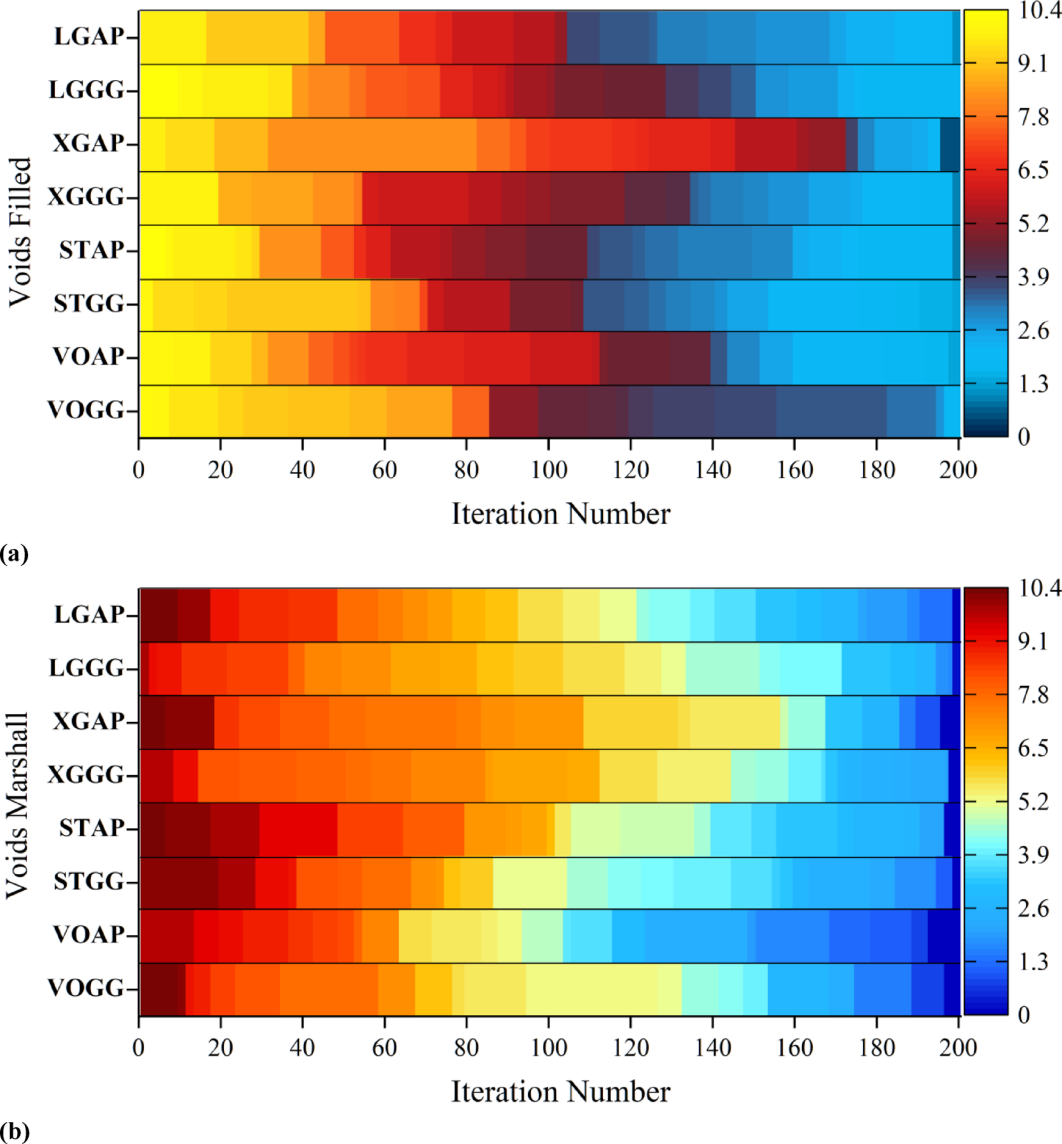
Heatmap illustrating the convergence behavior of the developed hybrid models based on RMSE values, (**a**) Voids Filled and (**b**) Voids Marshall.

Figure 8 presents heatmaps of RMSE values across iterations of different hybrid models, combining underlying ML models with optimization techniques. Underlying models include LightGBM (LG), XGBoost (XG), Stacking (ST), and Voting (VO), and optimization techniques include Artificial Protozoa Optimizer (AP) and Greylag Goose Optimization (GG). Heatmaps display the dynamics of RMSE values across successive iterations, revealing the patterns of convergence for the hybrid models. The heatmaps utilize a color scheme in which red/orange shades indicate higher values of RMSE, indicating poorer performance, and blue shades indicate lower values, indicating better performance. The x-axis represents the number of iterations of the optimization process, and the y-axis lists the hybrid models with different combinations of base models and optimization methods. The figure is divided further into two subfigures: (a) illustrates the Voids Filled convergence, and (b) illustrates the Voids Marshall convergence, enabling comparison of the performance of the models on these two target variables. The results depict significant patterns in converging behavior. Ideally, the heatmaps should become progressively cooler in color with increasing iterations, indicating that the models are converging and the RMSE is decreasing. The rate and consistency of the change vary across the different hybrid models. A comparison of color patterns across rows reveals which models achieve the lowest RMSE and exhibit the best converging behavior. Models that transition quickly to cooler shades and exhibit low values of RMSE across iterations demonstrate improved performance. One of the important points is the impact of optimization techniques on the performance of the models. Comparing models with the same underlying model but different optimizers, it is evident that the GG is better than the AP as the GG-based models have a bigger blue area towards the later iterations in the heatmaps, i.e., smaller values of RMSE and better optimization efficiency. Moreover, the behavior of convergence is different between Voids Filled and Voids Marshall, which suggests that some models and optimization techniques perform better on one target variable than on the other. The heatmaps also provide insight into the consistency and stability of models. Models with consistently low RMSE values across iterations tend to exhibit higher stability, while models with fluctuating RMSE values may be less stable. The implications of the optimization approach and model choice are significant.

### The evaluation result of the models

**Fig. 9:**
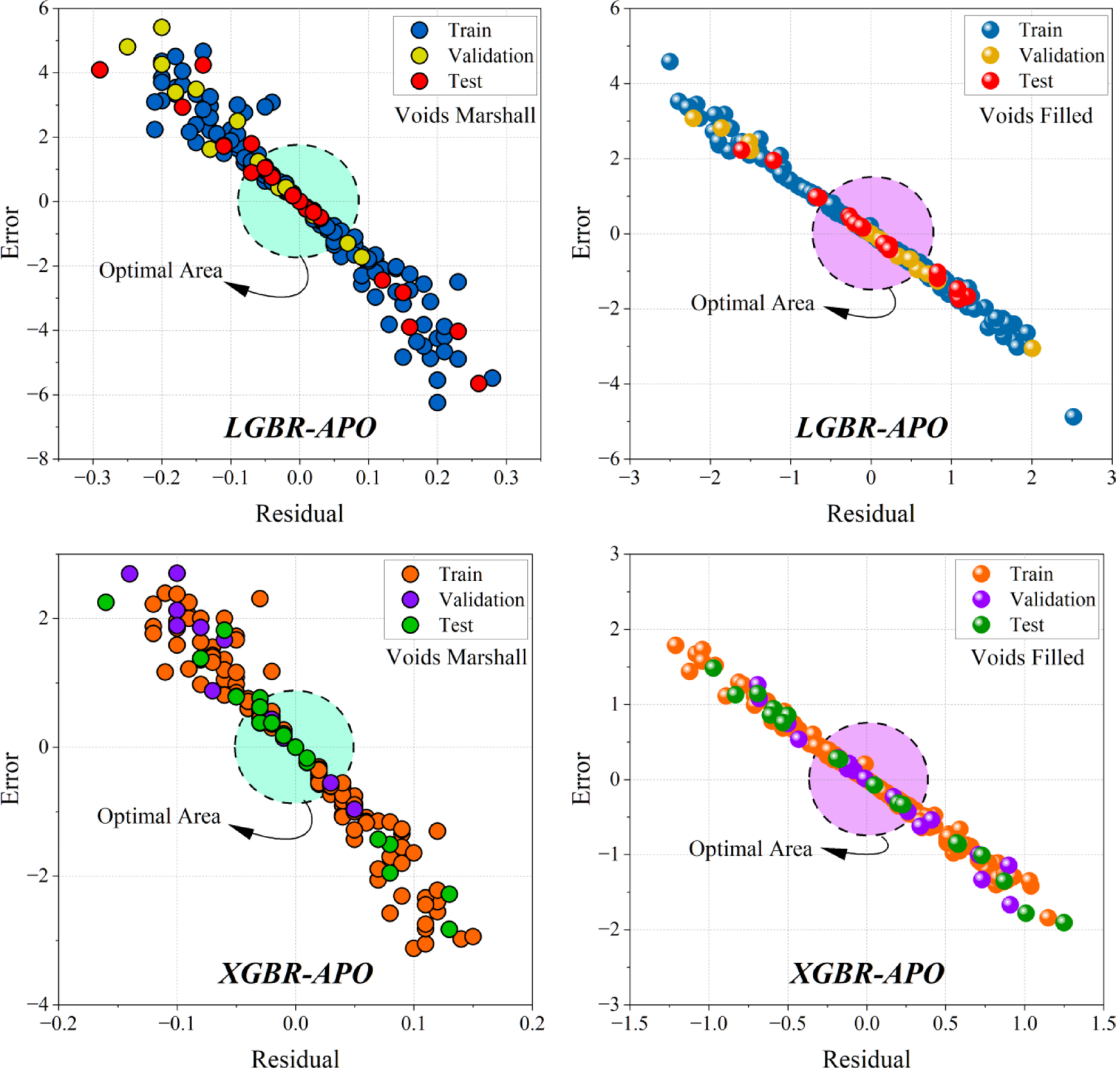

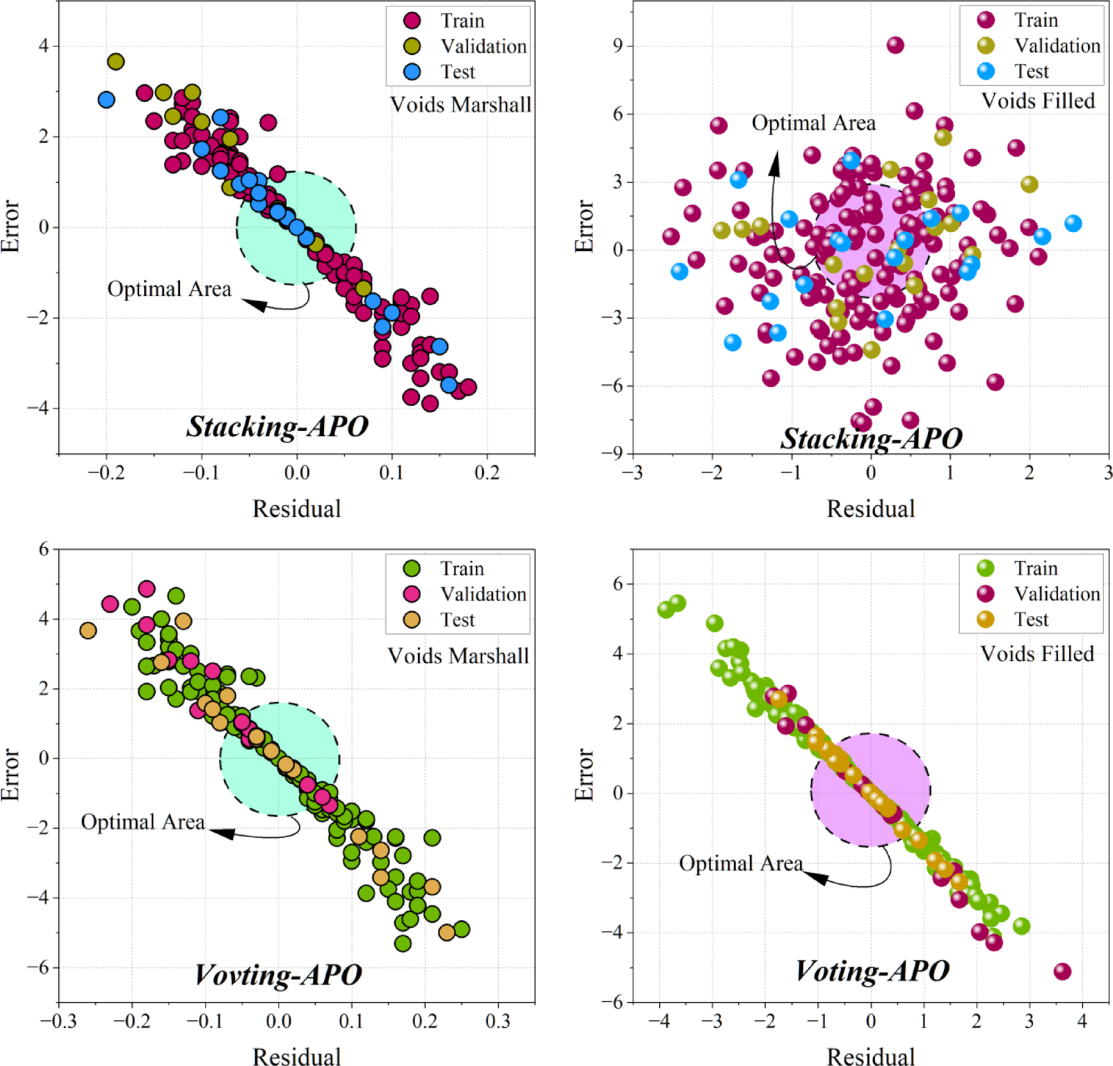
Evaluation of the model’s prediction accuracy for Voids, Marshall, and void-filled conditions using a Residual vs. Error plot. The “Optimal Area” refers to the region where the model yields minimal errors and residuals across the Train, Validation, and Test datasets.

Figure 9 provides a visual representation of the models’ accuracy in predicting the target variables. The closer the data points are to the origin (0,0), the better the accuracy of the model, with the Optimal Area being the area of optimal performance. When comparing the LGBR and XGBR models, it is possible to observe their relative performances per target variable. Both models exhibit a high linear correlation between residuals and errors, indicating good predictive ability. For the “Voids Filled” target, however, the XGBR model exhibits tighter clustering of data points in the optimal area, indicating better performance. The distribution of data points across the Training, Validation, and test datasets is also an important consideration when assessing model generalization. Ideally, all three data sets should be evenly distributed around the optimal area, ensuring uniform performance. The groupings of data points within the optimal area on all four graphs indicate that the models generalize well. A strong linear correlation between errors and residuals also suggests that the models reflect the patterns in the data. A departure from this correlation may be a sign of a flaw in the dataset or the model. The effectiveness of the APO is evident as most data points lie within the optimal area, indicating its efficiency in optimizing the models.

**Fig. 10:**
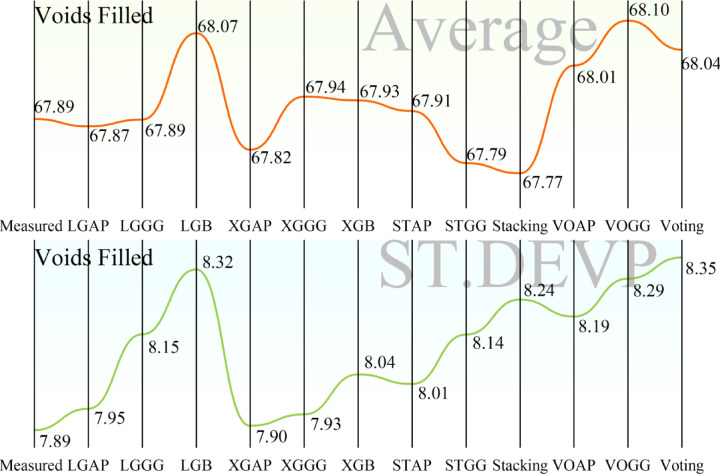
Comparison of developed models in predicting Voids Filled, showing both the average predicted value and the standard deviation of predictions.

Figure 10 presents two line charts comparing the performance of models in predicting “Voids Filled.” The higher chart is a plot of the average predicted values by each model, where the better scenario is when the predicted values are closer to the “Measured” value, i.e., higher accuracy. The lower chart is a plot of the standard deviation (ST.DEVP) of the forecasts, where a low standard deviation represents higher consistency and reliability of the model’s forecasts. Accuracy-wise, the graph at the top helps find the models whose mean values of what is being predicted approach the actual measured value. Graphically, LGB, XGB, Stacking, and Voting models correlate well with the measured values. The graph at the bottom helps find the reliability of the models by showing the standard deviation. The smaller the standard deviation, the higher the reliability of a model’s prediction. The models with the lowest standard deviation are XGB, Stacking, and Voting. Accuracy and consistency should be considered when determining the optimal model.

The ideal model should exhibit closely matched values between the predicted and measured values, as well as low variability in its output. From a visual inspection, the XGB, Stacking, and Voting models have performed well in both aspects. Among the models, XGAP has the least standard deviation, indicating its output is the most consistent. Additionally, its output values are closely comparable to the measured values, making it the best-performing model in Voids Filled, as shown in Fig. 10. This finding aligns with the broader research environment, where XGR, LGBR, Stacking, and Voting models were implemented and optimized using APO and GGO to enhance performance. The strong performance of stacking and voting further supports the research findings that ensemble methods tend to perform better predictively. The models were trained on well-selected, impactful features, which increased both efficiency and accuracy. Moreover, they were trained and tested on a dataset of approximately 200 asphalt samples to ensure practical applicability. The use of ML models, particularly XGBoost, helps reduce the time and cost associated with traditional asphalt performance testing.

**Fig. 11:**
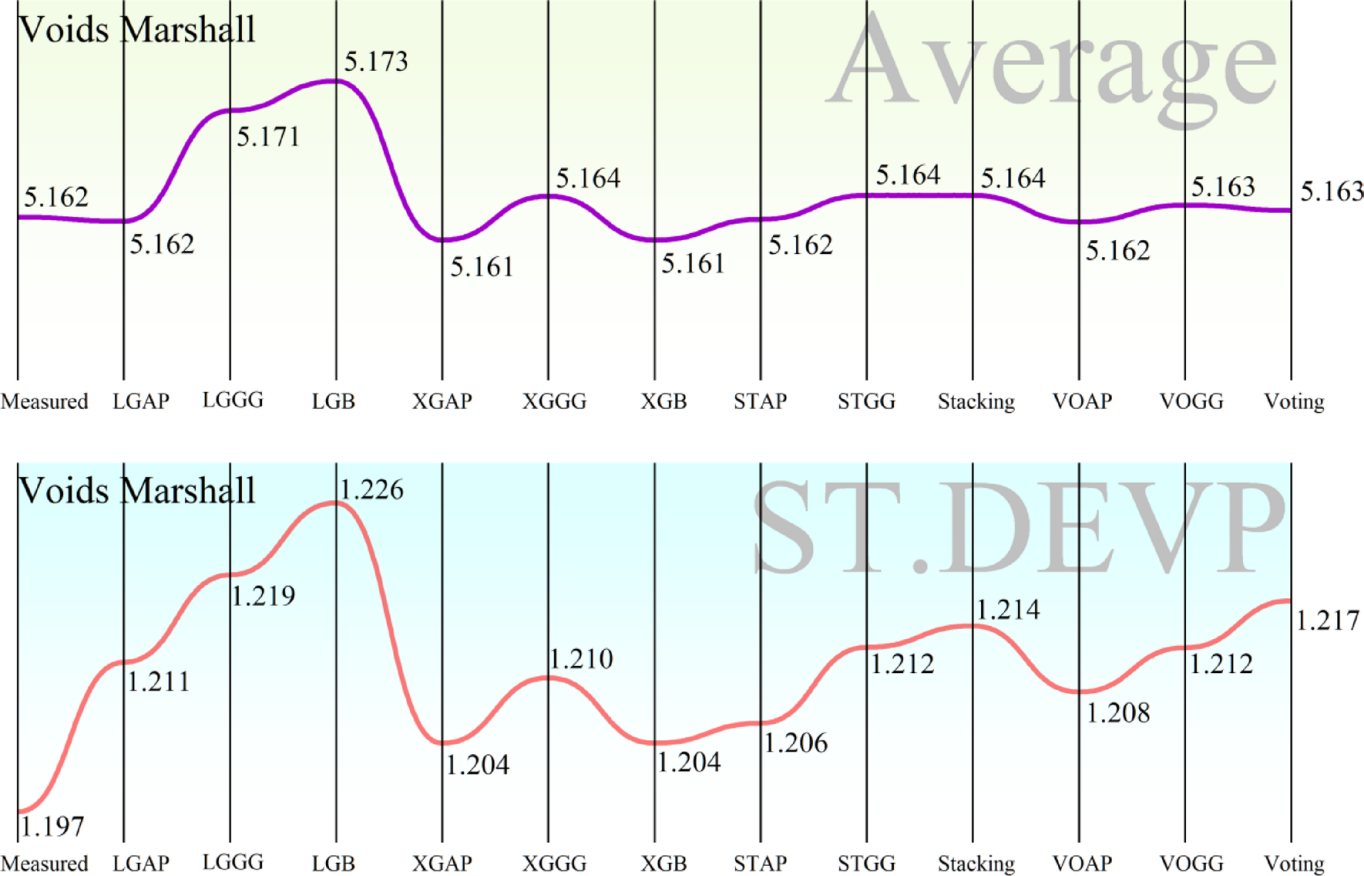
Comparison of developed models in predicting Voids Marshall, showing both the average predicted value and the standard deviation of predictions.

Figure 11 is organized in the same way as Fig. 10 but is directed towards the Voids Marshall target variable instead of Voids Filled. It is made up of two line graphs: the upper graph is the average predicted Voids Marshall values per model, and the bottom graph is the standard deviation (ST.DEVP) of the forecasts, which is a measure of the consistency of each model’s output. In terms of accuracy, the top graph is compared to the measured value, where, in the event of high accuracy, the predicted values should be highly similar to the measured value. From visual inspection, the models whose average predictions are closest to the measured value are XGBoost (XGB), Stacking, Voting, and LGAP. On the other hand, the bottom graph provides information on the consistency of the models by showing the standard deviation of the forecasts, where low values indicate stable and reliable outputs. Models with the smallest standard deviation include XGB, XGGG, STAP, Stacking, and Voting. For the performance evaluation of the models, the ideal models should be highly consistent (with low standard deviation) and highly accurate (closer to the measured value). Between the two graphs, XGB, Stacking, and Voting perform better. The XGB is perfect in both aspects, with very low proximity to the measured value and low standard deviation. The Stacking and Voting Methods also perform well, with LGAP having a highly accurate average predicted value but a higher standard deviation than XGB. Accuracy and consistency-wise, Stacking, XGBoost, and Voting models deliver the best overall performance. Of all the models, XGBoost is the most consistent across all predictions and is thus the best-performing model for predicting Voids Marshall, as shown in Fig. 11. These results complement the findings in Fig. 10, where XGBoost consistently performed well in predicting asphalt properties. The reliability and robustness of XGBoost in modeling asphalt properties are also reflected in the consistency of its performance across target variables. Additionally, the research findings validate the performance of ensembling techniques, such as stacking and voting, demonstrating their efficacy in terms of predictive accuracy.

### Attributes analysis

**Fig. 12:**
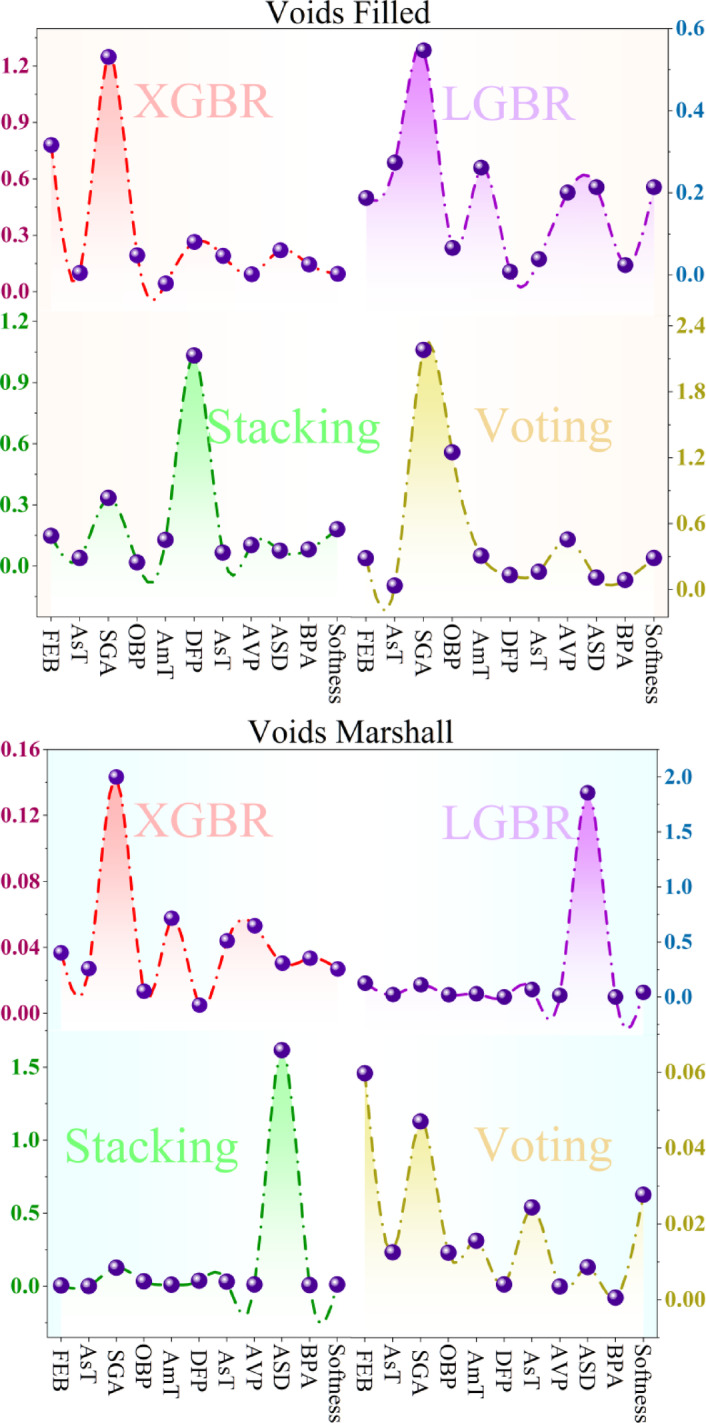
SHAP sensitivity analysis was performed on the models using the entire dataset utilized in the study.

Figure 12 presents SHAP values for each of the 14 features in the dataset, calculated for each of the four ML models, indicating the contribution of each feature to the predictive results of the models. Figure 12 is divided into two panels, with the upper half presenting SHAP values for Voids Filled and the lower half presenting SHAP values for Voids Marshall. Within both sections, the panels are again divided into four, corresponding to the four models: XGBR, LGBR, Stacking, and Voting. The x-axis on each plot represents the 14 features in the dataset, abbreviated to avoid confusion (e.g., FEB, Ast, OBP), and the y-axis indicates the SHAP values, which represent the size of each feature’s impact. The SHAP analysis highlights the most important features in both the models and target variables, with greater peaks in the plot corresponding to features having a greater impact on the forecasts. Comparing SHAP plots across models reveals differences in feature importance, as different models assign varying levels of importance to the same features. The comparison of SHAP values across Voids Filled and Voids Marshall also indicates that various features affect each target variable, further indicating that these properties depend on different factors of the asphalt mixture.

The plot helps identify the features that consistently have high SHAP values across models and target variables. Interestingly, both OBP (optimum bitumen percentage) and BPSM (bitumen percentage in asphalt mixture) appear to be highly relevant to both Voids Filled and Voids Marshall in all models. This is as expected in the dataset description, since features like OBP and BPSM, as well as aggregate properties like SGA, tend to have a significant impact on asphalt performance. Environmental factors, i.e., Ast and AmT, also have high SHAP values, indicating that they, too, play a role in influencing the predictions. The research highlights that certain properties, namely SGA and Ast, exhibit high variability, and SHAP analysis provides insight into the impact of these variables on the model’s predictions. If a highly skewed feature has a high SHAP value, it suggests that the model is sensitive to outliers in the feature. SHAP values make the model more interpretable by identifying the most significant features and their impact on the predictions, which is crucial in validating the model’s behavior. Key observations from Fig. 12 involve the dominance of OBP and BPSM, as they consistently exhibit high SHAP values, suggesting that both indeed have a significant influence on the target variables. Though variation in feature importance is present across models, consensus is achieved on the most significant features. SHAP values also suggest certain feature dependencies on Voids Filled and Voids Marshall, suggesting that different factors drive these asphalt properties. From a practical perspective, SHAP analysis verifies the importance of factors that other methods of selection, such as RFE, have highlighted, thereby further enhancing the reliability of the feature selection. SHAP values also add additional insights that make the model more interpretable, offering a better understanding of the decision-making process that occurs in the predictions.

The SHAP analysis not only identifies the most influential features but also provides insights into the physical and mechanical behavior of the asphalt mixtures. For instance, OBP and BPSM consistently exhibit the highest SHAP values across models and target variables, highlighting their critical role in volumetric properties. From an engineering perspective, higher OBP and BPSM generally reduce air void content in asphalt mixtures, improving cohesion but potentially lowering durability if excessive. Similarly, aggregate properties such as SGA significantly affect Voids Filled and Voids Marshall; higher aggregate density tends to reduce voids, enhancing structural stability. Environmental factors such as Ast and AmT also influence predictions, reflecting the temperature-dependent viscoelastic behavior of asphalt, where lower temperatures may stiffen the mixture and affect void distribution.

Furthermore, features exhibiting high variability or skewness, when associated with high SHAP values, indicate that the model is sensitive to extreme values of these parameters. This insight has practical implications: engineers can prioritize controlling OBP, BPSM, and aggregate properties during mixing and compaction to achieve desired volumetric characteristics. In summary, the SHAP results validate the engineering significance of the selected features, confirm the effectiveness of RFE-based feature selection, and provide actionable guidance for asphalt mixture design, highlighting which variables most strongly drive performance metrics such as Voids Filled and Voids Marshall.

### Discussion

The analysis carried out using Recursive Feature Elimination (RFE) has highlighted the importance of various features in predicting the asphalt properties, Voids Filled and Voids Marshall. The ranking of feature importance varied across models, revealing differences in the interpretation of features by XGBR and LGBR. The differences were further illustrated in the radially plotted importance, which emphasizes the varying importance each model attaches to features. The outcomes of the cross-validation reveal that both models have high predictive power, with XGBR consistently achieving a higher R^2^ than LGBR in most folds, indicating improved generalization. However, in some cases, LGBR presents competitive performance, particularly in Fold 3, revealing that its application could be effective in specific scenarios. The values of RMSE provide a further comparison, where XGBR illustrates a less fluctuating trend compared to LGBR, which shows higher variability across the folds. The improved performance of XGBR is particularly evident in Fold 1, where it illustrates the highest R^2^ (0.9660) and the lowest RMSE (1.48). Conversely, the worst performance of LGBR is in Fold 5, where it illustrates the lowest R^2^ (0.7647) and a high RMSE (11.74). The findings confirm XGBR’s preference on the grounds of overall consistency and predictive accuracy, with LGBR being an effective alternative where computational efficiency is required.

The heatmaps in Fig. 9 further validate the above results by showing the convergence of RMSE over iterations in different hybrid models. The interaction between Artificial Protozoa Optimizer (APO) and Greylag Goose Optimization (GG) is critical in influencing the performance of the models. Comparative analysis reveals that models involving GG exhibit improved convergence patterns and smaller RMSE over iterations, indicating that GG is a superior optimization method compared to AP. Additionally, the varying RMSE convergence between “Voids Filled” and “Voids Marshall” indicates that the performance of the models is target-dependent and hence requires a careful choice based on the specific property of interest. Figure 10 is a critical visualization of accuracy in the models, where the proximity of data points to the optimal area measures the efficacy of the models. XGBR exhibits a tighter cluster of data points in the optimal area in “Voids Filled,” further suggesting enhanced predictive power. The distribution of data across the Train, Validation, and Test sets further suggests that both models generalize well, as evidenced by a uniform distribution around the optimal area. The efficiency of APO is also evident, as it facilitates clustering within the optimal region, contributing to the reliability of the models. The line charts in Fig. 11 further reveal accuracy and consistency. The average values of XGBR, Stacking, and Voting models closely correspond to measured values, again suggesting accuracy. Additionally, the standard deviation analysis indicates the reliability of the models, with XGBR, Stacking, and Voting exhibiting the least variability. Such consistency is critical in practical situations where stable predictions are desired.

#### Comparison and implications

- Predictive accuracy: XGBR is better than LGBR in R^2^ in the majority of the folds and demonstrates improved clustering in the optimal regions.
- Error Stability: XGBR’s RMSE is smoother, and that of LGBR is more volatile; hence, XGBR is more stable.
- Feature Importance: The different rankings of features indicate that XGBR and LGBR have distinct interpretations of feature importance, which may be useful in understanding material properties.
- Efficiency Optimization: Hybrid models based on GG optimization tend to minimize RMSE more effectively, making them suitable for long-term predictive purposes.
- Model Generalization: XGBR and LGBR generalize well, with XGBR being better at maintaining high predictive power across data partitions.
- Hybrid Model Performance: Stacking and Voting models, when tuned with GG, provide a good balance of accuracy and consistency, making them good alternatives to standalone models.

## Conclusion

Predicting the performance of asphalt is a complex problem, as the durability and stability of the material are influenced by several factors. Traditional methods of assessment are time-consuming, costly, and require specialized devices; therefore, they are not suitable for large-scale or real-time applications. The present study overcomes the constraints by using computer learning methods to build foresight models that enhance managerial decision-making in asphalt quality appraisal. By incorporating sophisticated optimization and ensemble learning techniques, the present work not only addresses the challenges of numerous experiments on asphalt but also provides a performance evaluation method that is both economical and efficient. To do this, data from Iranian Ardabil roads, collected over 18 months, encompassing approximately 200 asphalt samples with 14 critical features, were studied. XGBoost and LightGBM models were applied to predict asphalt properties, and these predictions were made more accurate and precise through ensemble learning methods such as Voting and Stacking. Hyperparameter tuning using the Artificial Protozoa Optimizer (APO) and Greylag Goose Optimization (GGO) was also conducted to enhance the models’ efficiency and generalizability. Recursive Feature Elimination (RFE) was employed to identify the most significant features, which not only enhance the models’ interpretability but also reduce their computational complexity. The k-fold cross-validation method was employed to assess the model’s performance across various data partitions. The results indicate that the XGBR model was more efficient than LGBR regularly. Besides higher R^2^ values and lower RMSE, the errors also showed a uniform distribution across the different validation folds. XGBR achieved maximum R^2^ (0.9660) and minimum RMSE (1.48) in Fold 1, indicating better predictive ability. Although LGBR performed similarly well in some cases, e.g., in Fold 3, it exhibited higher variability in RMSE and is therefore less suitable for universal purposes. Ensemble learning methods, particularly Voting and Stacking, further improved predictive accuracy, with GG optimization being critical in optimizing model robustness and convergence. Heatmap visualizations and accuracy distribution plots confirmed that models with GG optimization performed better in terms of clustering around optimal prediction regions, justifying their effectiveness in minimizing RMSE and enhancing predictive reliability. These results have significant practical implications for road maintenance and construction. With predictive accuracy on the performance of asphalt, which is possible using ML, decision-making is proactive and less dependent on lengthy physical testing. Policymakers and engineers can utilize predictive models to design more durable road surfaces, select better materials, and plan maintenance more effectively. The results also demonstrate the potential of integrating hybrid optimization techniques into ML processes to further advance predictive modeling in civil engineering. Although the proposed XGBoost and LightGBM models, enhanced by ensemble techniques (Voting and Stacking), hyperparameter optimization (APO and GGO), feature selection, and sensitivity analysis, demonstrate strong predictive performance on the current dataset, there are some limitations. The dataset is relatively small (approximately 200 samples) and limited to a single city (Ardabil, Iran). Therefore, while internal validation indicates high accuracy, caution should be exercised when extrapolating these findings to other geographic regions, climatic conditions, or road types. Future studies should aim to include larger and more diverse datasets to enhance the generalizability and robustness of the predictive models. Future work can explore several promising directions. First, expanding the dataset to include diverse climatic and geographical conditions will enhance model generalization and robustness. Second, integrating additional features such as traffic load, binder type, or aggregate gradation could improve prediction accuracy. Third, developing real-time prediction systems using Internet of Things (IoT) sensors and edge computing would allow continuous monitoring of pavement performance. Fourth, incorporating deep learning architectures and hybrid metaheuristics could further boost nonlinear feature extraction and optimization efficiency. Finally, future research should focus on explainable AI (XAI) techniques to improve model transparency and facilitate trust and adoption in pavement engineering practice.

## Supplementary Information

Below is the link to the electronic supplementary material.

Supplementary Material 1

## Data Availability

The data supporting the findings of this study are provided in the supplementary materials.

## Abbreviations

XGB: Xtreme gradient boosting
APO: Artificial protozoa optimizer
NNDG: Non-nuclear density gauge
ML: Machine learning
ANN: Artificial neural networks
PG: Performance grading
Pba: Absorbed asphalt content
DSR: Dynamic shear rheometer
GBR: Gradient boosting regression
ICE: Individual conditional expectation
Aste: Asphalt temperature
AmTe: Ambient temperature
DFFP: Double-faced fracture percentage
AsD: Asphalt sample density
PVFB: Percentage of voids filled with bitumen
RMSE: Root mean square error
LGBM: Light gradient boosting machine
GGO: Greylag goose optimization
FWD: Falling weight deflectometer
CBR: California bearing ratio
DCT: Disk-shaped compact tension
Pbe: Effective asphalt content
SVR: Support vector regression
ETR: Extra tree regression
PSO: Particle swarm optimization
OBP: Optimum bitumen percentage
SGA: Specific gravity of aggregates
BPSM: Bitumen percentage in asphalt mixture
FEBWR: Filler-to-effective bitumen weight ratio
AVP: Aggregate void percentage
PVMS: Percentage of voids in the marshall sample
*RFE*: Recursive feature elimination
